# Reactive Oxygen Species-Responsive Polymer Nanoparticles to Improve the Treatment of Inflammatory Skin Diseases

**DOI:** 10.1021/acsomega.2c01071

**Published:** 2022-07-15

**Authors:** Heidi K. Noddeland, Pernille Kemp, Andrew J. Urquhart, Andreas Herchenhan, Klaus A. Rytved, Karsten Petersson, Louise B. Jensen

**Affiliations:** † Explorative Formulation & Technologies, LEO Pharma A/S, 2750 Ballerup, Denmark; ‡ Department of Health Technology, Technical University of Denmark, 2800 Kgs. Lyngby, Denmark; § Explorative Biology, LEO Pharma A/S, 2750 Ballerup, Denmark; ∥ In Vivo Biology & Safety, LEO Pharma A/S, 2750 Ballerup, Denmark; ⊥ Department of Pharmacy, University of Copenhagen, 2100 Copenhagen, Denmark

## Abstract

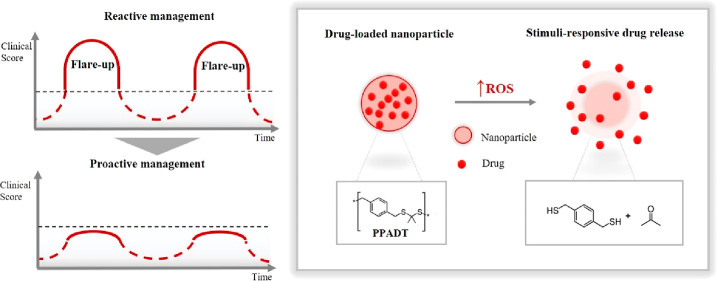

To improve the quality of life for people living with chronic inflammatory skin diseases, we propose a new treatment strategy by exploring a stimuli-responsive drug delivery system. Formulations designed by exploiting smart materials can be programmed to perform a specific action upon exposure to disease-related stimuli. For instance, increased levels of reactive oxygen species (ROS), especially the accumulation of hydrogen peroxide, can be utilized to differentiate between healthy and inflamed tissues. In this concept-proofing study, the polymer poly(1,4 phenyleneacetone dimethylene thioketal) (PPADT) was investigated for its ROS-responsive properties and potential to treat inflammatory skin diseases. PPADT nanoparticles were formulated by oil-in-water emulsification followed by solvent evaporation and characterized by size, zeta-potential, and release kinetic profiles. Release profiles revealed that the PPADT nanoparticles were sensitive toward elevated levels of ROS in an ROS-stimulus concentration (0.1–10 mM) and time-dependent manner (flare-up mimicked). The safety assessment proved that the PPADT polymer and the monomers generated by oxidation do not show any sign of being cytotoxic to fibroblasts and no mutagenic liabilities were observed. In conclusion, the PPADT polymer demonstrated to be a promising material for stimuli-responsive delivery of hydrophobic small molecules in the treatment of inflammatory skin diseases.

## 1 Introduction

Inflammatory skin diseases affect men and women in all ages around the world. The Global Burden of Disease study revealed that skin and subcutaneous disorders account for the fourth most common cause of nonfatal disease burden worldwide.^1^ These diseases are expressed in many forms from occasional rashes to chronic conditions such as atopic dermatitis, psoriasis, rosacea, and hidradenitis suppurativa.^2^ However, conditions such as acne, hives, and keloids exhibit a chronic inflammatory character in periods of life. Living with a chronic skin disease and treating it can be time-consuming and cumbersome. Beyond the itching, these conditions can cause pain and discomfort due to the redness, flakiness, and abnormal skin appearance.^3^ Some patients experience a tremendous impact on quality of life due to stigmatization, isolation, and mental health distress.^4^ Today, there is no cure for chronic skin diseases, and current treatments mainly revolve around management of symptoms and maintenance of remissions using topical products and/or selected oral antibiotics, antihistamines, corticosteroids, D-vitamin analogues, retinoids, and phosphodiesterase-4 (PDE4) inhibitors.^5^ Especially, topically administered products can be greasy, inconvenient to handle, and difficult to apply due to the advanced expression of the symptoms.^3,6^ Newly developed biological drugs have greatly improved the treatment efficacy for many patients by specifically targeting inflammatory hallmarks related to pathogenesis of dermatological diseases. However, this type of treatment is mostly prescribed to patients with severe inflammatory skin diseases.^5,7^ Moreover, the relapsing course of the disease requires frequent dosing and long-term treatment. Development of a new treatment strategy is needed to improve the quality of life for these patients. Potentially, a responsive treatment can be perceived as a functional therapy that can monitor the course of the disease and release drug when needed.^8−10^ Abnormally high levels of reactive oxygen species (ROS) have been associated with a range of diseases, including cancer, atherosclerosis, and several skin diseases with an inflammatory character.^11−17^

In this study, we investigated an ROS-sensitive polymer, poly(1,4 phenyleneacetone dimethylene thioketal) (PPADT), for its stimuli-responsive drug release properties and potential as a drug delivery vehicle in treatment of inflammatory skin diseases. Smart materials have been synthesized and used in formulation of drug delivery systems such as nanocarriers or hydrogels with ROS-sensitive moieties or linkers.^17−21^ The PPADT polymer has shown to be highly sensitive to elevated concentrations of ROS due to the thioketal linkages in the polymer backbone.^22−28^ In 2010, Wilson and co-workers published data showing that PPADT nanoparticles were able to deliver TNF-alpha-siRNA to sites of intestinal inflammation after oral administration. Using a murine model of ulcerative colitis, they demonstrated that the PPADT nanoparticles were able to withstand acid-, base-, and protease-catalyzed degradation seen in the gastrointestinal tract and selectively release siRNA in response to high levels of ROS at the target site.^22^ Data presented in Tang *et al.* demonstrated how the PPADT polymer could encapsulate stromal cell-derived factor 1α (SDF-1α). The nanoparticles were administered by intravenous infusion to mice with full-thickness skin defects. SDF-1α was successfully released and targeted to wounds in the presence of ROS.^23^ In another study conducted by Kim *et al.*, paclitaxel-loaded PPADT nanoparticles were tested with PC-3 prostate cancer cells. Paclitaxel is a well-known water-insoluble drug, and the study demonstrated that PPADT nanoparticles can be a new and promising material for intracellular delivery of insoluble drugs.^28^ However, these studies lack investigation of the ROS-sensitive potential of the PPADT nanoparticles in flare-up mimicked conditions and investigation of the release properties in fibroblast cell culture. Surprisingly, human dermal fibroblast (HDF) cells were challenged to produce significantly upregulated levels of ROS by changing the salt concentration in the medium (hypotonic challenge). This finding led to the foundation of a suitable and simple dermal model to test the ROS sensitivity of the PPADT nanoparticles.

To investigate the potential of PPADT as a drug delivery system for inflammatory skin diseases, ROS-responsive nanoparticles were prepared using the single emulsion solvent evaporation method. The physiochemical properties and drug release profile of the PPADT nanoparticles (Ø = ∼220 nm) were compared to poly(lactic-*co*-glycolic acid) (PLGA) nanoparticles (Ø = ∼243 nm). PLGA has been extensively studied and used as a polymer for conventional controlled release studies.^29,30^ Furthermore, PLGA is FDA-approved, is commercially available, and undergoes hydrolysis rather than oxidation.^31^ To make a proof-of-concept assessment of the PPADT nanoparticles, evaluation was conducted by physiochemical characterization, *in vitro* release testing (IVRT), safety testing, and performance in an HDF assay. The ROS stimulus applied throughout the proof-of-concept assessment was H_2_O_2_, which is one of the most stable forms of ROS and with a high abundance in the preinflammatory phase.^11^ Both exploratory (10 mM) and biologically relevant concentrations of H_2_O_2_ (100 μM) were investigated to unravel information about the ROS responsiveness and sensitivity of the ROS-sensitive PPADT nanoparticles in comparison to the ROS-insensitive PLGA nanoparticles.^10,11^ Nile Red (NR) was chosen as the model compound due to the molecular size and physiochemical properties, which render it relevant for modeling encapsulation and release of poorly water-soluble small molecules.^32^

## 2 Materials and Methods

### 2.1 Materials

PPADT was purchased from AGLYCON, Austria. A description of the synthesis of PPADT can be found in Section S1 in the Supporting Information. Upon receipt, PPADT was characterized by H^1^ NMR and size exclusion chromatography. The average molecular weight of PPADT was 4 kDa with a polydispersity index of 1.7, whereas the purity was found to be 96 mol %. PLGA R503H (*M*_w_. 24–38 kDa), NR (CAS: 7385-67-3), partially hydrolyzed poly(vinyl alcohol) (PVA) (4-88 EMPROVE), and hydrogen peroxide (35 wt % aqueous) were purchased from Sigma-Aldrich, Germany. Dichloromethane (DCM), ethyl acetate, ethanol, acetonitrile, 1,4-benzenedimethanethiol, and cellulose dialysis membrane (25 mm, 9 kDa cut-off) were purchased from Thermo Fisher Scientific Co., Ltd. unless otherwise stated. Massachusetts US. Dulbecco’s modified Eagle’s medium (DMEM), Dulbecco’s phosphate-buffered saline (DPBS, no calcium, no magnesium), Alexa Fluor cellular stains (phalloidin, hoechst, CellROX Green, CellROX Deep Red), 3-(4,5-dimethyl2-thiazolyl)-2,5-diphenyl-2*H*-tetrazolium bromide (MTT), and formaldehyde solution were purchased from Thermo Fisher Scientific, Denmark. Cell counting cassettes (Via1-Cassette) were purchased from ChemoMetec, Denmark.

### 2.2 Formulation of PPADT and PLGA Nanoparticles

The PPADT and PLGA nanoparticles were prepared by oil-in-water (o/w) emulsification followed by solvent evaporation. Briefly, 25 mg of polymer (PPADT or PLGA) was dissolved in 1 mL of DCM in a screw-cap glass vial to form the organic solution. The organic solution was vortexed, and the vial was left for 5 min to ensure that the polymer was completely dissolved. Meanwhile, the water solution was prepared, wherein 8 mL of PBS containing 1 wt % polyvinyl alcohol (PVA) was added to a separate screw-cap glass vial. The organic solution was transferred to the water solution and vortexed thoroughly for 1 min to obtain the o/w solution. Thereafter, the o/w solution was emulsified using a probe sonicator (40% amplitude, continuous output, probe tip 3.2 mm) for 5 min in an ice bath. The organic solvent was evaporated from the nanoparticle suspension in vacuum using a rotatory evaporator (BUCHI Rotavapor R-215). The placebo nanoparticles were centrifuged at 12,000 rpm for 5 min and washed three times with PBS to remove residual PVA. Finally, the nanoparticles were suspended in PBS and stored at 5 °C, protected from light.

NR-loaded PPADT and PLGA nanoparticles were formulated following the same procedure as for the placebo nanoparticles, except that 0.5 mg of NR was dissolved in 0.5 mL of DCM and added to the organic solution before vortexing. The NR-loaded nanoparticles were recovered from the unencapsulated NR by centrifugation and washed as previously described. The chemical structure of PLGA and NR can be seen in Figure S1A,B, respectively.

### 2.3 Particle Size and Zeta-Potential

The hydrodynamic size, polydispersity index (PDI), and zeta-potential were measured using dynamic light scattering (DLS), Malvern’s Zetasizer Nano. For each formulation, three independent size measurements were obtained. To measure the zeta-potential, the nanoparticle solution was diluted 1:10 in purified water. Thereafter, the prepared sample was added to a folded capillary cell (gold-plated electrodes, DTS1060c). A zeta-cap was added to seal both ends of the capillary channel. Both hydrodynamic size and zeta-potential measurements were conducted at 25 °C.

### 2.4 Encapsulation Efficiency

The encapsulation efficiency is used to estimate how much of the drug is successfully entrapped within the PPADT and PLGA nanoparticles. The nonencapsulated amount of NR was determined in the discarded supernatant obtained in the recovery phase of the PPADT and PLGA nanoparticles using high-pressure liquid chromatography (HPLC, Waters ACQUITY UPLC H-class system, Massachusetts, US). The encapsulation efficiency was calculated using eq 1.
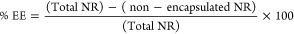
1

### 2.5 H_2_O_2_ Exposure Study

The exposure study was designed to investigate the influence of H_2_O_2_ on the nanoparticle size distribution. Two concentrations of H_2_O_2_ were used: 0.1 and 10 mM. PPADT and PLGA nanoparticles were mixed with H_2_O_2_ solutions at the following timeslots: 0 min, 15 min, 60 min, 4 h, 8 h, 24 h, 48 h, and 120 h. Incubation of these samples took place on a vortex table (50 rpm) at 37 °C. The vials were covered during incubation to avoid exposure to light. After exposure to 0.1 and 10 mM concentration of H_2_O_2_, the nanoparticle solution was analyzed with DLS.

### 2.6 Release Study and Modeling

**Figure 1:**
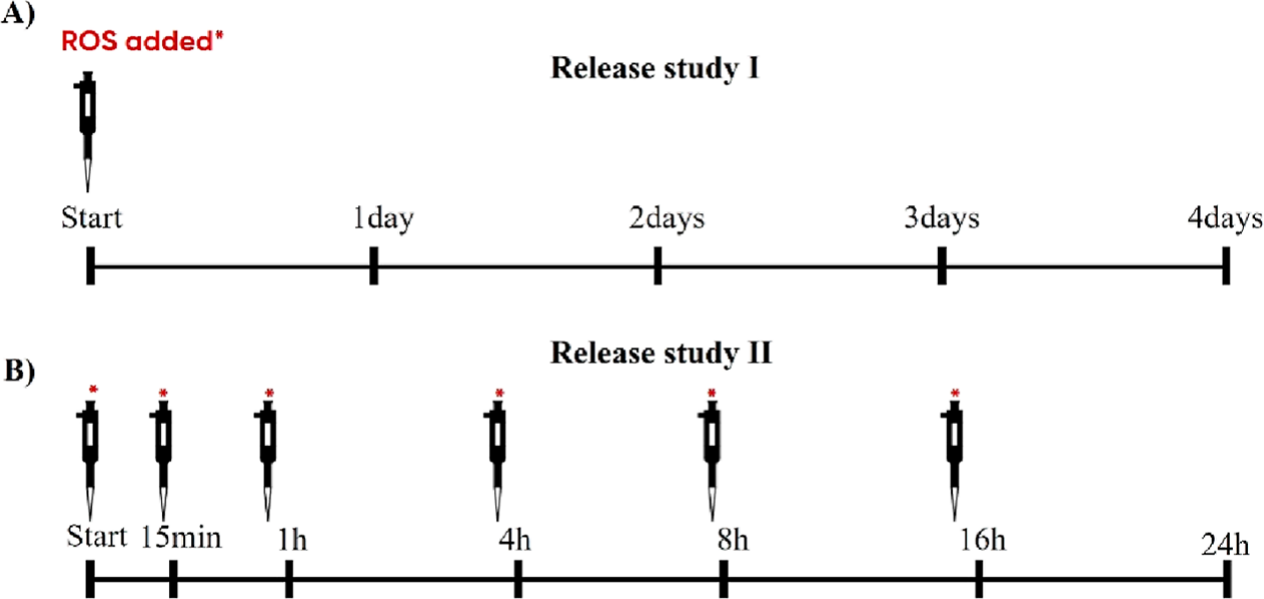
Overview of the sampling time, length of study, and addition of ROS stimulus (0.1 and 10 mM) for release study I (A) and for release study II (B). In release study II, the ROS stimulus was added 30 min prior to sampling after the 1 h measurement.

To study the NR release of PPADT and PLGA nanoparticles, an IVRT experiment using Franz diffusion cells was conducted. A cellulose membrane (Mw. cutoff: 9 kDa) was inserted to separate the donor and the receptor chamber. Prior to the experiment, the membranes were immersed in PBS overnight. 2 wt % HP-β-CD (*M*_w_. 1.396 kDa) was added to the receptor medium to avoid the unbound NR to bind to the IVRT tubes, ensure sink conditions, and at the same time increase the solubility of NR. The release chamber was equipped with a magnetic stir bar and set to 800 rpm. The temperature was maintained at 37 °C. During the experiment, the Franz diffusion cells were protected from light exposure to avoid breakdown of H_2_O_2_. The first release study was conducted over a period of 4 days where samples were withdrawn at predefined timeslots: 0, 24, 48, 72, and 96 h (Figure 1A). In this study, PPADT and PLGA nanoparticles were exposed to 1 mL of 10 mM H_2_O_2_ and controls were exposed to PBS. The kinetic release data obtained in the first 24 h were fitted to the following kinetic release models: zero order, Higuchi, and Korsmeyer–Peppas (Figure S2).

In the second release study, the PPADT and PLGA nanoparticles were tested applying a fresh stimulus at different timeslots over a course of 24 h (Figure 1B). Different concentrations of H_2_O_2_ were used: 10, 0.1 mM, and control (PBS). Samples with a volume of 0.5 mL were withdrawn from the release medium at predefined timepoints and replenished with fresh medium.

### 2.7 HPLC—Quantification of NR

The concentration of NR was quantified using HPLC (Waters ACQUITY UPLC H-class system, Massachusetts, US). Data collection and processing were conducted using the software Empower. The separation was carried out on a reverse-phase C18 column (Waters Acquity BEH, 1.7 μm, 2.1 mm × 50 mm). The mobile phase consisted of the acetonitrile/water gradient going from a 5:95 ratio to a 95:5 ratio which was maintained for 1 min and back to 5:95. The total run time was 7 min, and the flow rate was maintained at 0.5 mL/min. NR was monitored between 210 and 500 nm. For quantification, NR was detected by absorption at 265 nm (peak: 264.9 nm) and 310 nm (peak: 308.6 nm). Samples of 5 μL were injected into the column. All HPLC measurements were performed with a column temperature of 40 °C. The retention time was 1.27 min for NR.

### 2.8 HDF Assay

#### 2.8.1 Cell Handling and Staining

The HDF cells were grown in T-175 culture flasks at 37 °C in a 5% CO_2_ humidified incubator. The cell growth medium (DMEM + 10% FBS + 1% Penstrep) was changed three times per week. For experimental purposes, HDF cells were harvested at passage 2–4 with a confluence of 80–90%. The HDF cells were seeded in a ×96 well plate with a density of 4.0 × 10^3^ cells/well and allowed to attach to the bottom 24 h prior to experimental start.

Four different stains were applied: hoechst, phalloidin, CellROX Green, and CellROX Deep Red. Two CellROX stains were used to investigate cell stress at two wavelengths, 488 nm for the CellROX Green stain and 647 nm for the CellROX Deep Red stain. This was carried out to obtain more robust data and to avoid interference with the model drug, NR. All samples and conditions were tested in triplicates. Hoechst staining and phalloidin staining were applied as a default to all samples, CellROX Green in one of the triplicates, and CellROX Deep Red in the other one. Live staining was performed for both the CellROX stains, whereas the cells were fixated with 4% formaldehyde when carrying out the hoechst and phalloidin staining.

#### 2.8.2 Stress-Induced Generation of ROS

A stress-induced ROS assay was developed to simulate the increase in ROS seen during inflammation. The HDF cells were plated in ×96-well plate as described previously and challenged with hypotonic medium (10 mM sodium phosphate buffer, pH = 7.4) in different ratios (1:20, 1:10, and 1:5) and H_2_O_2_ in different concentrations (0.01, 0.1, and 10 mM). The cells were challenged for 60 min or 120 min in an incubator at 37 °C. Development and validation of this assay for increased stress-induced generation ROS by HDF cells were validated using a high-content fluorescence imager (Operetta CLS, Perkin Elmer). Staining was performed as described in the previous section.

#### 2.8.3 Release Performance in Stress-Induced ROS Assay

Release performance of PPADT and PLGA nanoparticles loaded with NR was tested in the stress-induced ROS assay by applying 50 μL of nanoparticle solution to the challenged cells. The nanoparticles were incubated for 60 min and analyzed with the high-content fluorescence imager. Staining was performed as described in the previous section, except that CellROX staining was excluded in order to avoid measuring the intensity generated by ROS. Consequently, the intensity could be solely related to the NR release.

### 2.9 Safety Evaluation

#### 2.9.1 *In Silico* Investigation

An in silico investigation was performed by testing PPADT and the monomer 1,4-benzenedimethanethiol in the software packages DEREK Nexus (version 6.1.1) and SARAH Nexus (version 3.0.0) The software is from Lhasa, Ltd, Leeds, UK. DEREK predicts mutagenicity and skin sensitization potential using rule-based expert software. SARAH predicts mutagenicity using statistical modeling software.

#### 2.9.2 Mutagenicity Testing

The mutagenic potential of PPADT and its breakdown products was tested in an Ames test performed at Gentronix Ltd, Manchester, UK. The compounds were tested in the TA98 strain (frameshift mutations) and the TA100 strain (base-pair substitutions). The monomer 1,4-benzenedimethanethiol was tested in the presence and absence of metabolic activation with the rat S9 fraction. The PPADT polymer was only tested in the absence of metabolic activation due to its poor solubility in DMSO and tetrahydrofuran (THF). The test was carried out in a microplate format. A modified version of the Ames test using a fluctuation method in which a liquid bacteria culture was used instead of agar plates was used. Incubation and selection of revertant colonies were performed in 384-well microplates, obtaining a colorimetric endpoint.

### 2.10 Statistical Analysis

Results are presented as mean ± SD with minimum three samples in parallel for *in vitro* release studies and for HDF cell experiments. The statistically significant difference between groups was determined by one-way analysis of variance (ANOVA). Hypothesis tests of comparison were determined using Student’s *t*-test, wherein *p*-values of less than 0.05 were accepted as significant (*p-value < 0.5 and **p-value < 0.01). All statistical measures were conducted using GraphPad Prism 8 Inc.

## 3 Results

### 3.1 Formulation and Characterization

Placebo and NR-loaded PPADT and PLGA nanoparticles were successfully formulated by the single emulsion solvent evaporation method and characterized by DLS, pH measurements, and encapsulation efficiency. The placebo nanoparticles had a hydrodynamic size of 220 ± 4 nm and 243 ± 2 nm for PPADT and PLGA, respectively. For the NR-loaded nanoparticles, the hydrodynamic size was 246 ± 3 nm for PPADT and 302 ± 3 nm for PLGA. The encapsulation efficiency was found to be 9.4% for PPADT nanoparticles and 10.7% for PLGA nanoparticles. For both the PPADT and the PLGA nanoparticles, the zeta-potential was close to zero, with a pH between 7.1 and 7.2 (Table 1).

**Table 1:** Hydrodynamic Size, PDI, and Zeta-Potential Measured with DLS^a^

formulation	size (d. nm)	PDI	zeta-potential (mV)	pH	% EE
Placebo					
PPADT	220 ± 4	0.09 ± 0.02	–1.45 ± 0.1	7.2 ± 0.1	
PLGA	243 ± 2	0.12 ± 0.01	–0.76 ± 0.7	7.1 ± 0.3	
*NR*					
PPADT	246 ± 3	0.09 ± 0.02	–1.53 ± 1	7.2 ± 0.1	9.4 ± 2.5
PLGA	302 ± 3	0.11 ± 0.01	–0.51 ± 0.4	7.1 ± 0.5	10.7 ± 1.9

a The encapsulation efficiency (% EE) for NR-loaded nanoparticles is indicated in the column to the right.

**Figure 2:**
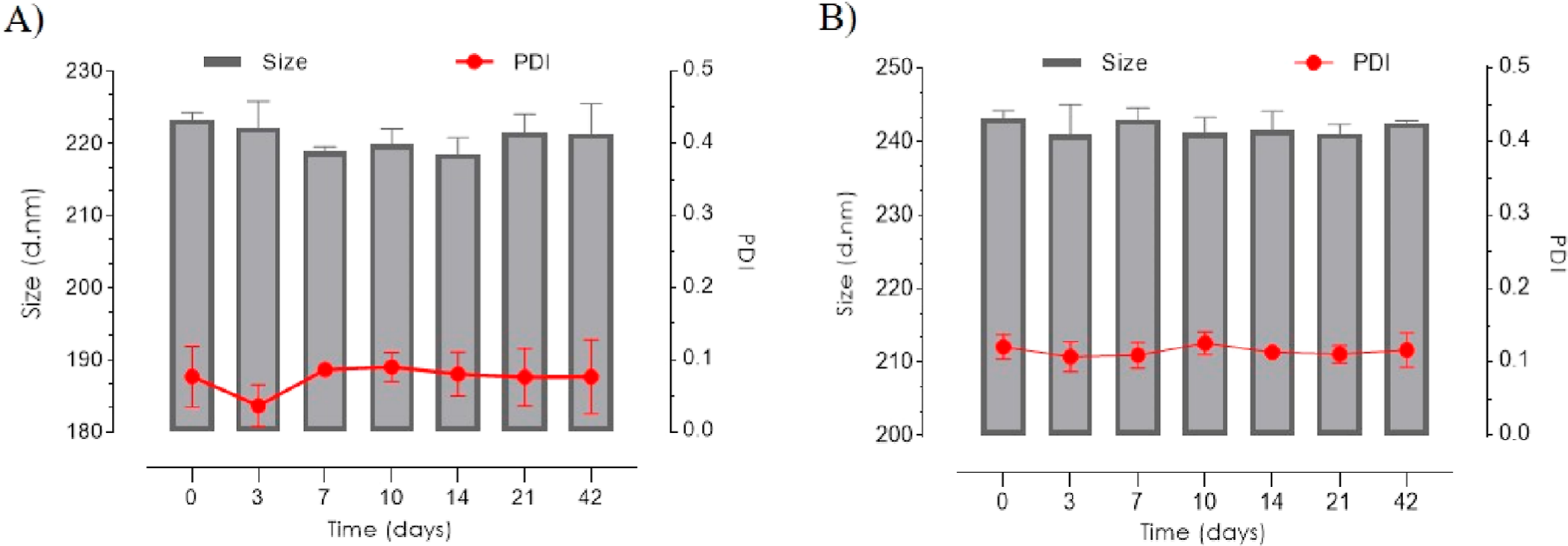
Stability DLS measurements of placebo nanoparticles suspended in PBS for 6 weeks at 5 °C. (A) Hydrodynamic size and PDI for PPADT nanoparticles. (B) Hydrodynamic size and PDI for PLGA nanoparticles.

The stability of the nanoparticles was monitored over a course of 6 weeks. During this period, the PPADT and PLGA nanoparticles maintained a stable hydrodynamic size, with PDI measurements below 0.1 (Figure 2A,B).

**Figure 3:**
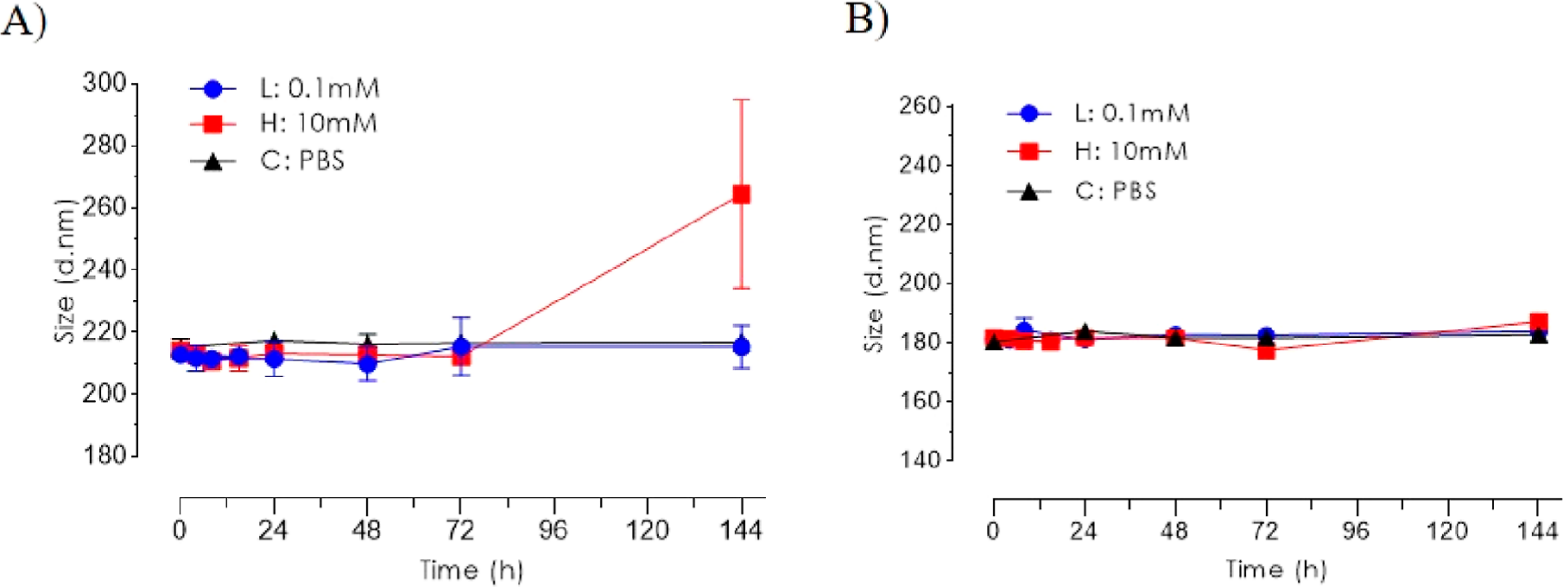
H_2_O_2_ exposure and hydrodynamic size change nanoparticles suspended in PBS at 37 °C. L = low (0.1 mM H_2_O_2_), H = high (10 mM H_2_O_2_), and C = control (PBS). (A) Hydrodynamic size and PDI for PPADT nanoparticles. (B) Hydrodynamic size and PDI for PLGA nanoparticles.

For investigation of the ROS responsiveness and degradability, the nanoparticles were incubated with 0.1 and 10 mM H_2_O_2_ aqueous solution. Notably, PPADT nanoparticles showed significant changes in size and PDI measurements after 72 h, when exposed to 10 mM H_2_O_2_ (Figure 3A). For all other size measurements, both for PPADT and PLGA (Figure 3B), PDI measurements were below 0.12.

### 3.2 ROS-Responsive Release

**Figure 4:**
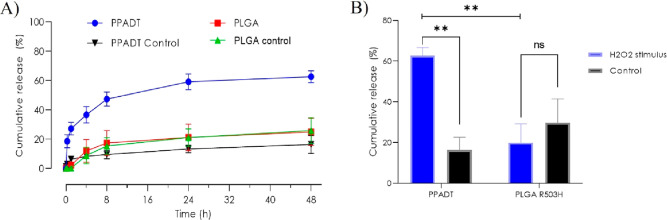
Release testing for NR-loaded PPADT and PLGA when exposed to 10 mM H_2_O_2_ and PBS (control). (A) Release profile of NR over a period of 48 h. (B) Cumulative release of NR after 48 h incubation.

The cumulative release of NR reached 60% for PPADT nanoparticles when exposed to 10 mM H_2_O_2_ after 24 h. The release profile for PPADT showed an initial burst release within the first few hours, and after 24 h, the cumulative release began to equilibrate (Figure 4A, PPADT). In comparison, only 20% NR release was seen for PLGA nanoparticles after 24 h incubation with 10 mM H_2_O_2_ (Figure 4A, PLGA). Notably, there was a significant difference for the H_2_O_2_-exposed PPADT nanoparticles and the control (PBS) but not for the PLGA nanoparticles (Figure 4B).

**Figure 5:**
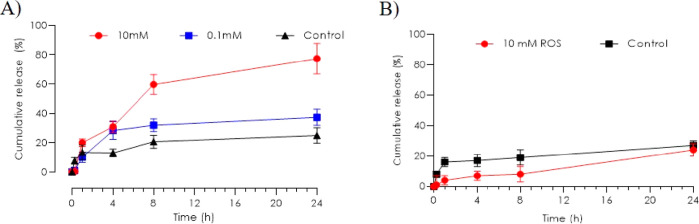
Flare-up mimicked release of NR from PPADT and PLGA nanoparticles. The H_2_O_2_ stimulus was added 30 min prior to sampling. (A) Release profiles for PPADT when exposed to 10, 0.1, and 0 mM H_2_O_2_ (control). (B) Release profile for PLGA when exposed to 10 and 0 mM H_2_O_2_ (control).

To mimic the relapsing course of inflammatory skin diseases, a flare-up mimicked drug release study was conducted, wherein the nanoparticles were exposed to several doses of H_2_O_2_ for a period of 24 h. The H_2_O_2_-exposed PPADT nanoparticles demonstrate a significantly higher NR release compared to the control (Figure 5A) and the H_2_O_2_-exposed PLGA nanoparticles (Figure 5B). Two different H_2_O_2_ concentrations were applied to investigate the sensitivity of the PPADT nanoparticles. It can be seen that the release of NR from the PPADT nanoparticles was both H_2_O_2_ concentration-dependent and time-dependent. Increasing the H_2_O_2_ concentration from 0.1 to 10 mM induced a significantly higher NR release after 4 h exposure. After 24 h, the cumulative release was four times higher for the 10 mM H_2_O_2_-exposed PPADT nanoparticles compared to the control.

In the flare-up mimicked environment, the release profile reached a ∼20% higher cumulative release compared to the release profile when H_2_O_2_ was added once. In comparison, PLGA nanoparticles were exposed to 10 mM H_2_O_2_ with the same exposure frequency and time period. The cumulative release was ∼30% for both the H_2_O_2_-exposed PLGA nanoparticles and the control with no significant difference between the two conditions.

### 3.3 Stress-Induced Generation of ROS by HDFs

**Figure 6:**
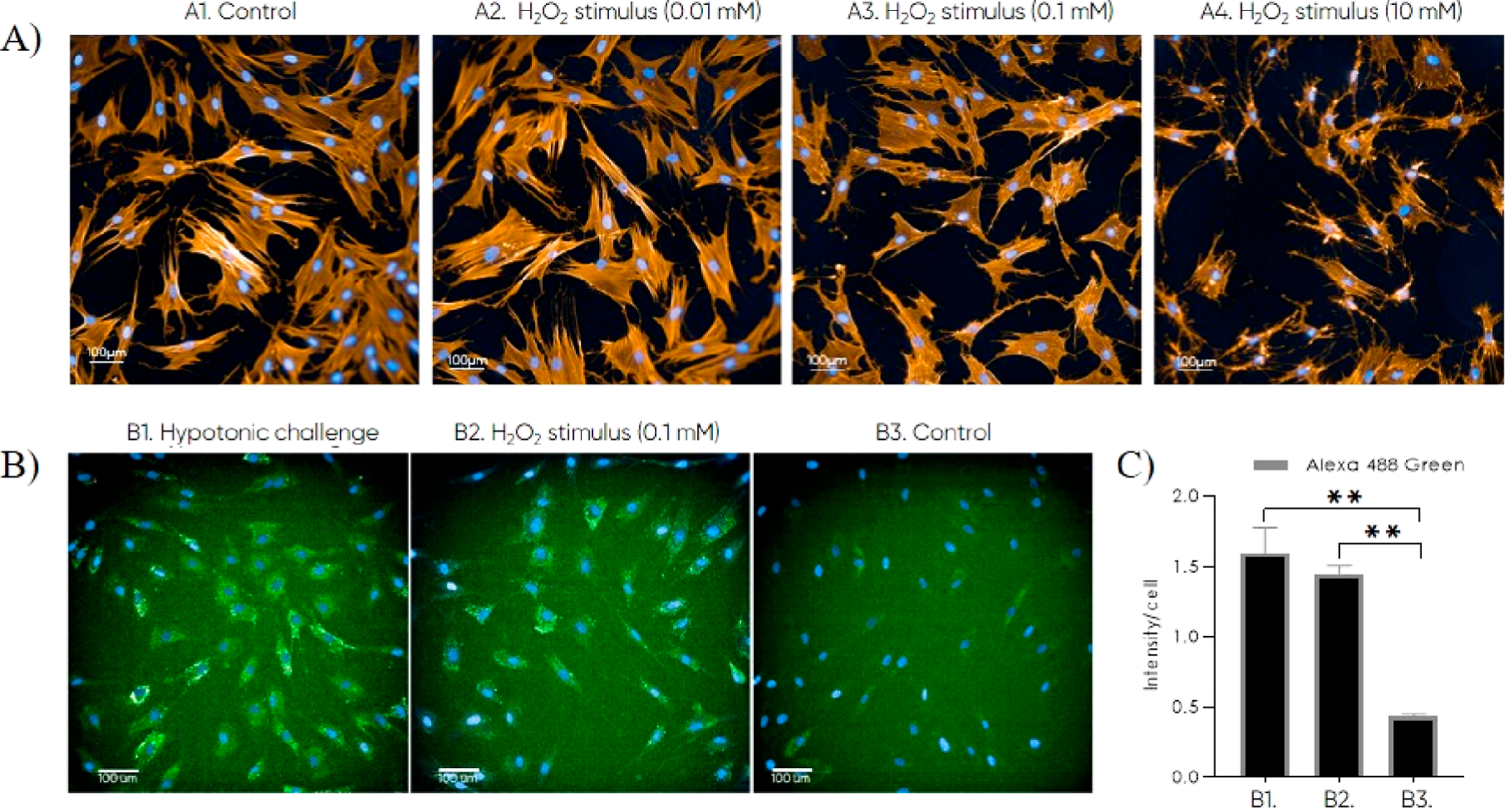
Detection of ROS in fibroblast cells after 2 h challenge with H_2_O_2_ (0.01–10 mM) or hypotonic challenge. Blue staining: hoechst, orange staining: phalloidin, and green staining: CellROX Green. (A) Fluorescent images of HDF cells exposed to 0.01, 0.1, and 10 mM H_2_O_2_ to investigate the morphology of the cells. (B) Fluorescent intensity per cell when the fibroblasts were hypotonically challenged, exposed to 0.1 H_2_O_2_ and DMEM growth medium (control). (C) Abundance of ROS (intensity/cell) for hypotonic challenge [B(1)], H_2_O_2_ stimulus [B(2)], and control [B(3)].

HDF cells were stressed to produce increased concentration of ROS by the hypotonic stimulus and H_2_O_2_ exposure to enable a biological relevant ROS assay to investigate drug release. Hoechst staining and phalloidin staining were added to all wells to enable visualization of the cell nucleus and actin morphology, respectively. Green and red CellROX stainings were added in separate wells, and no CellROX staining was added in the remaining. The staining strategy made it possible to differentiate between ROS generation, NR release, and intensity differences due to noise. As a safety measure, the PPADT and PLGA nanoparticles, placebo and NR-loaded, did not show any morphological changes or any significant upregulation in ROS production compared to the control. The statistical conclusion was made by conducting a one-way ANOVA on the data obtained in the green channel, revealing that the green CellROX staining captured the ROS production better than the red CellRox staining. Interestingly, the hypotonic stimulus did not change the morphology, whereas the H_2_O_2_ exposure did at higher concentrations (Figure 6A). Thus, the hypotonic stimulus represents a promising new way of inducing higher ROS production in a simple fibroblast assay [Figure 6B(1)]. The upregulation of ROS was measured as signal intensity/cell, wherein both the hypotonic stimulus and the H_2_O_2_ exposure generate significantly higher intensity compared to the control (Figure 6B,C).

### 3.4 *In Vitro* Nanoparticle Performance in ROS-Induced Fibroblast Assay

**Figure 7:**
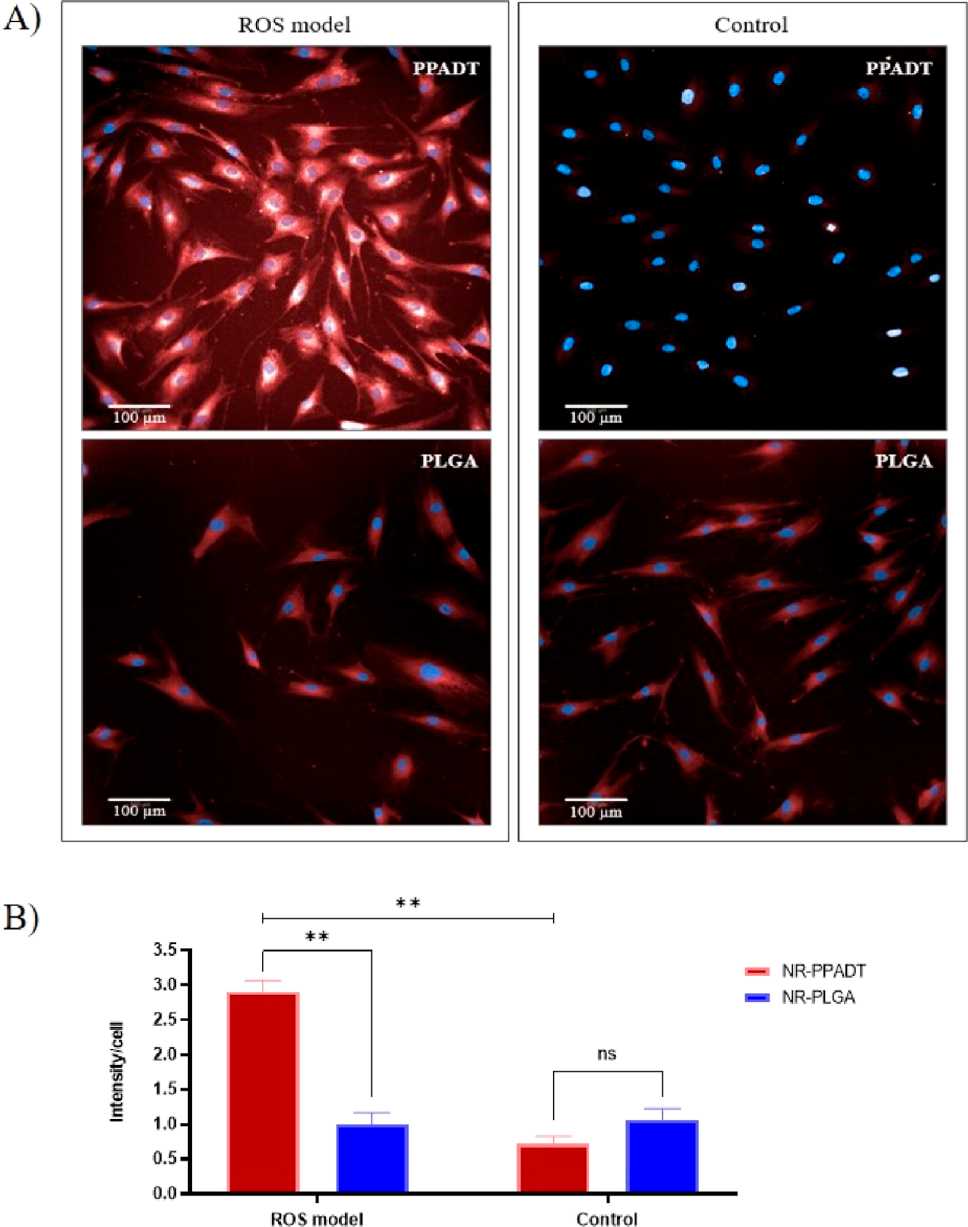
Release performance of PPADT and PLGA nanoparticles in ROS-induced fibroblast assay (1 h incubation). The NR signal was collected at 488 nm (Alexa 488 channel). (A) Fluorescent images of fibroblasts showing the NR release performance for PPADT and PLGA nanoparticles under challenged (ROS-induced model) and control conditions. (B) Fluorescence signal per cell for NR-loaded nanoparticles in the ROS-induced model and under control conditions.

The drug release of NR-loaded PPADT and PLGA nanoparticles was evaluated in both ROS-induced fibroblasts and in unstimulated control fibroblasts for 1 h. In the ROS-induced fibroblast assay, a high NR signal was observed for PPADT and a significantly lower signal was observed for PLGA (Figure 7A). In the unstimulated control fibroblast assay, a very low NR signal was seen for PPADT nanoparticles, and a slightly higher NR signal was seen from PLGA nanoparticles (Figure 7A). Here, the well with the lowest NR signal of the PLGA control was chosen, but the overall NR signal obtained as triplicates provides a signal that was not significantly different from the NR signal in the PPADT control. However, in the ROS-induced model, only PPADT nanoparticles proved to induce a stronger NR signal. The NR signal for PPADT in the ROS model was significantly higher compared to the signal for the PLGA nanoparticles and close to threefold higher than the control conditions for PPADT. In contrast, there was no significant increased NR release in the ROS model for PLGA and no significant difference between the PLGA and PPADT under the control condition (Figure 7B).

### 3.5 In Silico Toxicity Testing for Mutagenicity and Skin Irritation Potential

PPADT and its breakdown products, 1,4-benzenedimethanethiol and acetone, were investigated in two in silico models: SARAH NEXUS, a statistic software tool for mutagenicity prediction and DEREK NEXUS, knowledge-based software that predicts mutagenicity, skin irritation potential, and other endpoints. Following processing in the two software programs, the compounds were classified as listed in Table 2, and 1,4-benzenedimethanethiol was classified as moderate skin sensitizers in both models.

**Table 2:** *In Silico* Testing for Mutagenicity and Skin Irritation

	in silico end point (test system)		
compounds	mutagenicity (SARAH)	mutagenicity (DEREK)	skin sensitization (DEREK)
PPADT	no alerts	no alerts	moderate sensitizer
1,4-benzenedimethanethiol	no alerts	no alerts	moderate sensitizer
acetone	positive	no alerts	no alerts

SARAH NEXUS software classified acetone as being a potential mutagen. This is not confirmed in the literature where acetone has proven nongenotoxic in various genetic toxicity assays.^35^

### 3.6 *In Vitro* Mutagenicity Testing

The highest concentration of PPADT tested was 4.4 μg/mL (1.1 μM) and 15.4 μg/mL (90 μM) for the monomer (Table 3). No revertants above threshold were observed when testing the PPADT polymer and the monomer (1,4-benzenemethanethiol) for both strain TA98 and TA100 in the Ames test. The monomer was tested successfully both with and without metabolic activation (+S9).

**Table 3:** Assessment of Mutagenic Potential for PPADT and the Monomer Using Ames MPF Assay^a^

		results			
		strain TA98		strain TA100	
compound	highest conc. (μg/mL)	–S9	+S9	–S9	+S9
PPADT	4.369	√		√	
monomer (1,4-benzenemethanethiol)	15.36	√	√	√	√

a The strains used were TA98 (frameshift mutation) and TA100 (base-pair substitution). Metabolic activation (+S9) was only applied when testing the monomer. (√) no observed revertants above threshold.

The first data points were pure diluent (100% THF), and the monomer concentration was increased for each data point until the maximum concentration was reached. The red dotted line indicates the threshold in which a critical amount of revertants have back-mutated to the prototrophic state (Figure S3). In conclusion, no critical fraction of revertants was found for the monomer and the PPADT polymer for both strains, TA98 and TA100.

## 4 Discussion

### 4.1 Preparation and Characterization of PPADT Nanoparticles

Stimuli-responsive drug delivery can be achieved by utilizing the discrepancy in ROS levels between healthy and inflamed tissues to improve the treatment of chronic inflammatory skin disease.^5,11^ Among the varieties of endogenous stimuli exploited so far (temperature, pH, glutathione, proteases, and glucose),^7,8^ ROS has motivated researchers to design responsive materials that can respond to disease-controlled concentrations (*e.g.*, cancer). In general, stimuli-responsive drug delivery systems have been challenged with poor sensitivity, rendering them irrelevant for biological applications. However, due to the abnormally high increase in biological ROS levels seen during inflammation, ROS-responsive drug delivery systems bring promising new opportunities for flare-up regulated controlled drug release. The PPADT nanoparticles formulated here show characteristics (size, PDI, zeta-potential, and encapsulation efficiency) comparable to the existing literature and in addition possess a promising size stability profile.^22,26,28^ However, a change in size distribution was observed for PPADT nanoparticles after 72 h when exposed to 10 mM H_2_O_2_, which indicates that the structure of the PPADT nanoparticles likely has changed due to degradation. To validate the structural change of the PPADT nanoparticles, the morphology must be investigated, which can be carried out by electron microscopy imaging (*e.g.*, transmission electron microscopy). Furthermore, this can be supplemented with 1H NMR and gel permeation chromatography to monitor the molecular weight distribution when exposed to ROS.^24,25^ If intended for long-term application as a depot, the encapsulation efficiency plays a crucial role. Depending on the solubility and potency of the drug, a relatively high amount of drug needs to be loaded into the depot, as multiple doses must be stored to reduce the dosing frequency. To increase the loading, nanoprecipitation-based microfluidic methods show great potential. Xu and co-workers demonstrate that the controllable microfluidic flow-focusing method can load doxorubicin into PLGA nanoparticles with an encapsulation efficiency of 88%.^36^

### 4.2 Release Study

Symptoms caused by chronic inflammatory skin diseases tend to occur with a relapsing course.^37,38^ To improve the spatiotemporal release of small molecules (*e.g.*, anti-inflammatory drugs) in accordance with the initiation of flare-ups, the sensitivity of the ROS-responsive nanoparticles is a crucial parameter. Interestingly, PPADT nanoparticles proved to be ROS-responsive in both a stimulus concentration and time-dependent manner. The PPADT nanoparticles exposed to H_2_O_2_ (0.1 and 10 mM) did show a significant higher cumulative release compared to the PPADT nanoparticles without the H_2_O_2_ stimulus (control). Prior, it has been verified that H_2_O_2_ concentrations as high as 0.1 mM can be found under inflammatory conditions.^10,11^ There was a significant difference in the cumulative release (>50%) after 24 h between the exposed PPADT nanoparticles and the control. It was found that the cumulative release increased further to 80% after 24 h in the flare-up mimicked study. Thus, it can be confirmed that PPADT nanoparticles showed an ROS-responsive release that was dependent on the dynamics of the H_2_O_2_ concentration in the surrounding environment. These results were significantly different for the PLGA nanoparticles, which confirmed to be ROS-insensitive.

In the H_2_O_2_ environments, the release of NR followed the Korsmeyer–Peppas model (*R*^2^ = 0.97 and *n* = 0.502) for the PPADT nanoparticles, which indicates that more than one release mechanism was involved.^39^ For polymeric nanoparticles, the Korsmeyer–Peppas model suggests that the polymeric chains rearrange, which can be caused by swelling or erosion of the surface, while at the same time, the diffusion process results in the time-dependent anomalous effect.^39,40^ However, the release profile for PPADT nanoparticles in the absence of H_2_O_2_ has the best fit to the Higuchi model (*R*^2^ = 0.962), which describes sustained release from a matrix. The release mechanism in this model is diffusion-controlled and can also be used to explain drug leakage over time, which can be supported by fitting of these data to the Korsmeyer–Peppas model (*R*^2^ = 0.901 and *n* = 0.421). Here, it can be read from the n-value that the release mechanism for PPADT nanoparticles without the stimulus is based on diffusion. Similar, PLGA nanoparticles demonstrate a release profile that can be fitted to the Higuchi model with and without H_2_O_2_ (*R*^2^ = 0.952). For the intended use as a functional depot, both a diffusion-controlled and a stimuli-responsive burst release in combination could be favorable to both initiate and maintain the therapeutic on-demand effect.

### 4.3 Fibroblast Studies

The purpose of fibroblast study was to investigate release behavior of PPADT nanoparticles in an environment with biologically relevant concentrations of ROS. Several methods have been used to challenge skin cells to produce increased levels of ROS, such as UV light, low pH, addition of serum amyloid A (5–15 μg/mL), IR irradiation, and addition of ROS species.^41−43^ Silverberg and co-workers used different concentrations of H_2_O_2_ to produce increased levels of ROS in fibroblasts, which was initially attempted here.^44^ Surprisingly, we found that changing the tonicity of the growth medium also challenged the cells to produce increased levels of ROS. Challenging the cells with H_2_O_2_ (0.1 and 10 mM) also increased the ROS levels. However, the morphology of the cells changed drastically when using H_2_O_2_, compared to challenging the cells for the same period with hypotonic medium. Interestingly, the change in tonicity lead to a threefold increase in the ROS signal, which may form the basis for a future ROS-induced fibroblast model, as the morphology of the cells was unaffected.

Here, the ROS-induced fibroblast model could be used to investigate the NR release performance for both the PPADT and the PLGA nanoparticle systems. The NR-loaded PPADT and PLGA nanoparticles were tested and compared to controls wherein no nanoparticles were added and to nonstimulated control cells with and without NR-loaded nanoparticles. The intensity/cell can be directly linked to the presence of NR since no CellROX staining was added to these test wells. The outcome of the study verifies an ROS-responsive release from the PPADT nanoparticles with biologically relevant sensitivity. Release of NR from the PLGA nanoparticles was independent of the ROS environment.

### 4.4 Safety Assessment

For polymers that are indented to be used as injectable depot formulations, it is crucial to investigate if the polymer itself or the breakdown products of the polymer can be cytotoxic or mutagenic. The Ames test showed that the PPADT polymer and the monomer were not able to cause production of revertants above threshold, meaning that no mutagenic liabilities were detected. The PPADT polymer, nanoparticles, and monomer were also tested in a HDF cell assay. It was found that the cell number and the morphology of the exposed cells did not change compared to the controls. Prior, PPADT nanoparticles have shown to have a minimal effect on cell proliferation on bone marrow mesenchymal stem cells and a promising cytotoxicity profile. An *in vivo* evaluation was conducted in mice where clinical measures and histological examination proved that the PPADT nanoparticles had a good biological compatilibliy.^23^ PPADT nanoparticles have also been evaluated on A549 and RAW264.7 cells and had no adverse effects on the cell viability.^24^ However, the in silico modeling classified PPADT and its monomer as a moderate skin sensitizer. This finding was based on the similarity to other thiols that have proven to elicit skin sensitization responses in *in vivo* models for skin sensitization. The mechanism is speculated to involve the reaction of the thiol group with skin proteins forming a hapten.^33,34^

Combining the outcome from the Ames test, the fibroblast studies, and in silico testing, it can be concluded that PPADT as a nanoparticle drug delivery system can be evaluated as nonmutagenic, with no cytotoxicity concerns but with the potential to act as a moderate skin sensitizer. As this was an in silico finding that was based on the similarity to other thiols, the finding should be further evaluated in an *in vitro* assay such as the “direct peptide reactivity assay” or the local lymph node assay.^45,46^

## 5 Conclusions

The PPADT polymer demonstrates relevant ROS-responsive features and performance, which was confirmed by the increased release of NR from the PPADT nanoparticles in the flare-up mimicked release study and in the ROS-induced fibroblast assay. The validation of the ROS-responsive release indicates that PPADT would be promising for use as a stimuli-responsive drug delivery system in treatment of inflammatory skin diseases. The ROS responsiveness of the PPADT nanoparticles was clearly concentration-dependent with a biologically relevant sensitivity, which certainly makes PPADT a promising candidate for use in on-demand delivery of hydrophobic small molecules. Moreover, the PPADT particles showed a promising size stability profile, and neither the PPADT particles nor the breakdown products did cause any mutagenic concerns.

## Supporting Information Available

Chemical structure of the PLGA polymer and NR; overview of the kinetic modeling of the release data generated in release study I (24 h) fitted to Higuchi, Korsmeyer–Peppas, and zero-order release models; and results obtained in the Ames test for the monomer tested on strain TA98 with and without metabolic activation (S9) (PDF)

## Abbreviations

DCM: dichloromethane
DLS: dynamic light scattering
DMEM: Dulbecco’s modified Eagle’s medium
FBS: fetal bovine serum
FDA: Food and Drug Administration
HFD: human dermal fibroblast
HP-β-CD: 2-hydroxypropyl-beta-cyclodextrin
HPLC: high-pressure liquid chromatography
IVRT: *in vitro* release testing
NMR: nuclear magnetic resonance
NR: Nile Red
PDE4: phosphodiesterase-4
PDI: polydispersity index
PLGA: poly(lactic-*co*-glycolic acid)
PPADT: poly(1,4 phenyleneacetone dimethylene thioketal)
PVA: polyvinyl alcohol
RNA: ribonucleic acid
SDF-1α: stromal cell-derived factor 1α
siRNA: small interfering ribonucleic acid
TNF: tumor necrosis factor
