# Supplementary material for: Reactive Oxygen Species-Responsive Polymer Nanoparticles to Improve the Treatment of Inflammatory Skin Diseases
Source: ACS Omega. 2022 Jul 15;7(29):25055–65. doi: 10.1021/acsomega.2c01071 (PMC9330180; doi:10.1021/acsomega.2c01071)
Supplement: Supplementary file 1 — ao2c01071_si_001.pdf [file ao2c01071_si_001.pdf]

Supporting Information

Reactive Oxygen Species - Responsive Polymer Nanoparticles to Improve the Treatment of Inflammatory Skin Diseases

Heidi K. Noddeland[a,e], Pernille Kemp[a*], Andrew J. Urquhart[b], Andreas Herchenhan[c], Klaus A. Rytved[d], Karsten Petersson[a] and Louise B. Jensen[a]*

[a]Explorative Formulation & Technologies, LEO Pharma A/S, 2750 Ballerup, Denmark

[b]Department of Health Technology, Technical University of Denmark, 2800 Kgs. Lyngby, Denmark

[c]Explorative biology, LEO Pharma A/S, 2750 Ballerup, Denmark

[d]In vivo biology & safety, LEO Pharma A/S, 2750 Ballerup, Denmark

[e]University of Copenhagen, Department of Pharmacy, 2100 Copenhagen, Denmark

S1. Synthesis of PPADT

Description from the manufacturer (AGLYCON, Austria):

1,4-Benzendimethanethiol (7.5 g, 44.04 mmol) was added to the solution of 2,2-dimethoxypropane (4.6 g, 44.17 mmol) and PTSA (0.026 g, 0.137 mmol) in 275 mL of anhydrous toluene and 25 ml ethyl acetate in a 1 L two-neck flask equipped with a distillation head and magnetically stirred for 1 h. Then, the heating bath was heated to 100°C followed by the addition of 2,2-dimethoxypropane (4.6 g, 44.17 mmol) in 220 mL of anhydrous toluene and 10 ml ethyl acetate at the rate of 0,3 mL/min for 12 h.

The reaction was magnetically stirred for additional 5 days at 120 °C bath temp. The clear brown reaction mixture was poured slowly in 2 L cold cyclohexane to precipitate the polymer as a brown sticky mass.

This material was dissolved in 10 ml DCM and precipitated a second time in 1L cold cyclohexane. The brown material was transferred in a round bottomed flask and dried on the rotavapor at 60 °C bath temp. and 1 mbar for 3 hours, to obtain a brown brittle foam.

Yield: 3.0 g

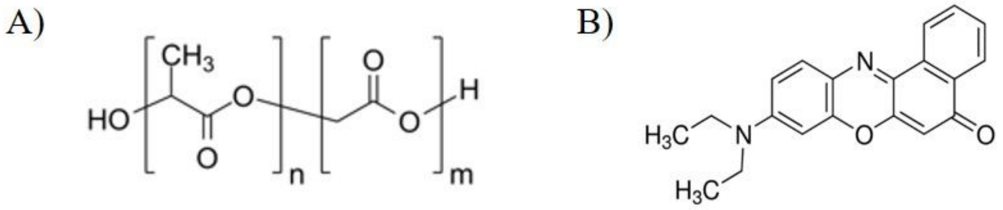

Figure S1. A) Chemical structure of PLGA R503H. B) Chemical structure of Nile red.

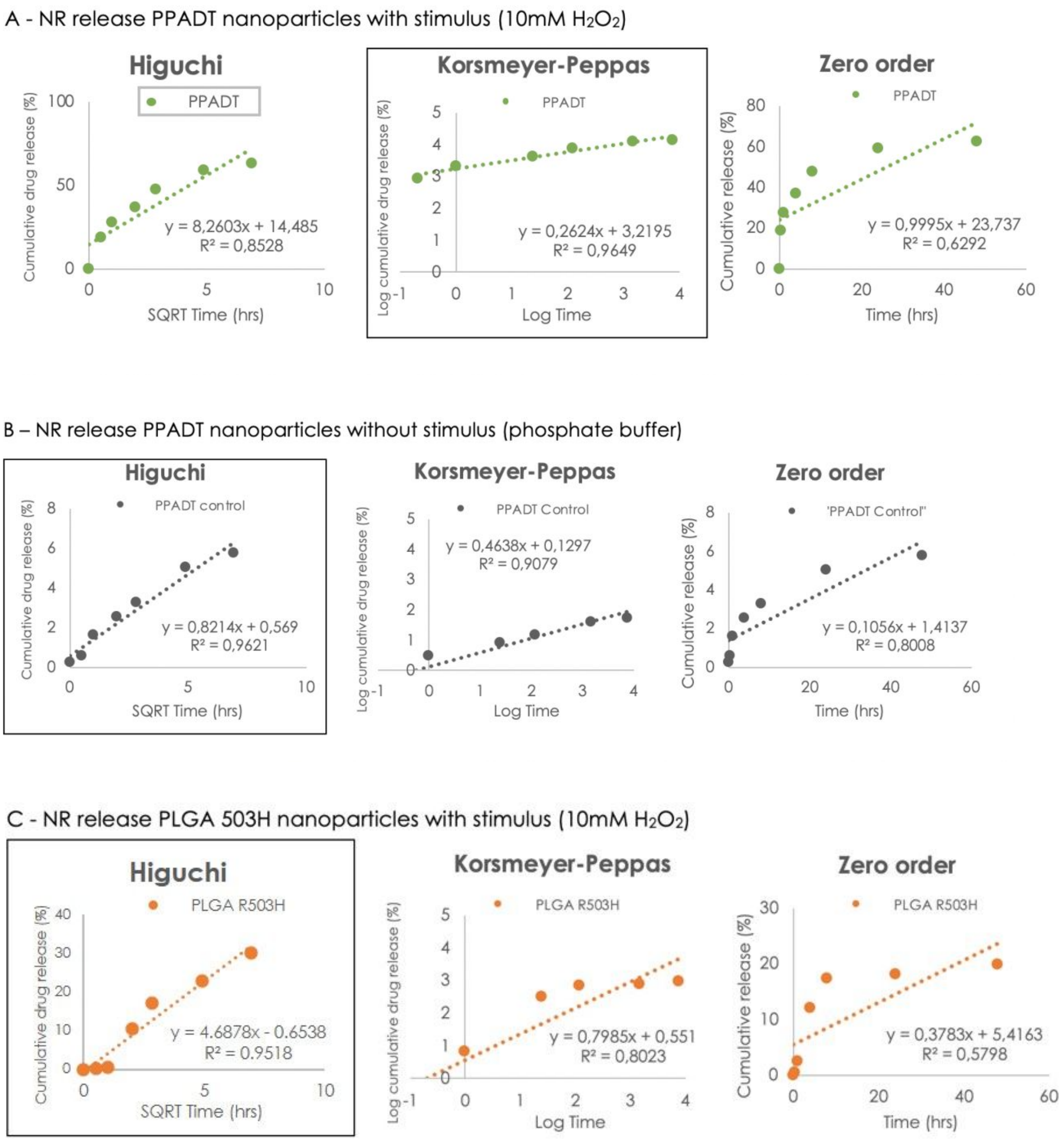

Figure S2. Kinetic modeling of release data generated in release study I (24 h) fitted to the Higuchi, Korsmeyer-Peppas and Zero order release model. A) PPADT nanoparticles exposed to H_2O_2 10mM. Korsmeyer-Peppas n-value = 0.502. B) PPADT particles exposed to pure PBS. Korsmeyer-Peppas n-value = 0.421. C) PLGA nanoparticles exposed to 10mM. Korsmeyer-Peppas n-value = 0.373.

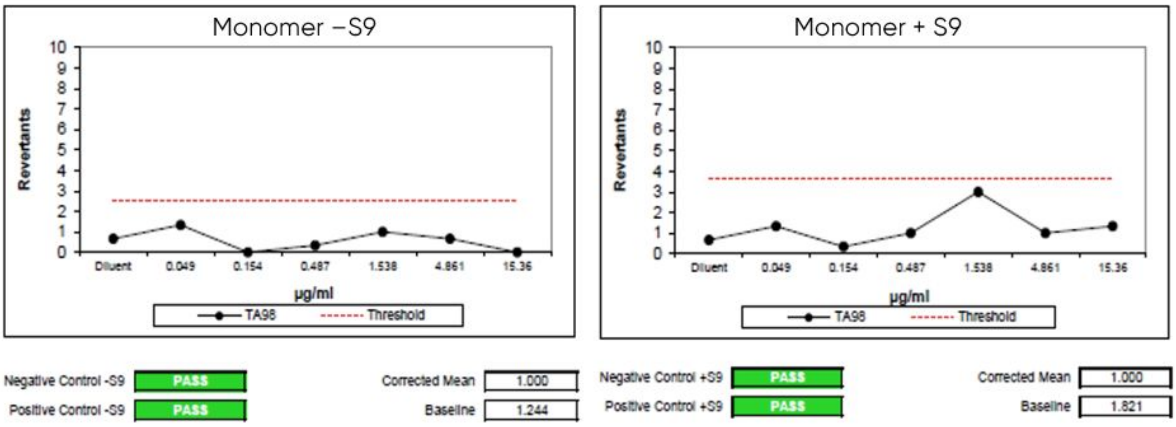

Figure S3. Results for the monomer tested in the Ames MPF assay on strain TA98 with and without metabolic activation (S9).
